# Ozone induces autophagy by activating PPAR*γ*/mTOR in rat chondrocytes treated with IL-1β

**DOI:** 10.1186/s13018-022-03233-y

**Published:** 2022-07-16

**Authors:** Panpan Sun, Weicheng Xu, Xu Zhao, Cong Zhang, Xiaowen Lin, Moxuan Gong, Zhijian Fu

**Affiliations:** 1 Department of Pain Management, The Second Hospital of Shandong University, Jinan, 250033 People’s Republic of China; 2 Department of Orthopedics, Shandong Provincial Hospital Affiliated to Shandong First Medical University, Jinan, 250021 People’s Republic of China; 3 Department of Anesthesiology, Shandong Provincial Hospital Affiliated to Shandong First Medical University, Jinan, 250021 People’s Republic of China; 4 Department of Pain Management, Shandong Provincial Hospital, Cheeloo College of Medicine, Shandong University, Jinan, 250021 People’s Republic of China; 5 Department of Pain Management, Shandong Provincial Hospital Affiliated to Shandong First Medical University, Jinan, 250021 People’s Republic of China; 6 Department of Anesthesiology, The Second Hospital of Shandong University, Jinan, 250033 People’s Republic of China

**Keywords:** O_3_, Osteoarthritis, PPARγ/mTOR, Autophagy, Inflammation

## Abstract

**Background:**

Osteoarthritis (OA) is the main cause of older pain and disability. Intra-articular injections of ozone (O_3_) commonly have been found to have antioxidative and anti-inflammatory effects to reduce pain and improve function in knee osteoarthritis. It has been reported that reduced autophagy in chondrocytes plays an important role in the development of OA. This study aimed to probe the role of O_3_ on the autophagy in chondrocytes treated with IL-1β.

**Methods:**

Primary chondrocytes were isolated from Wistar rats cartilage within 3 days. The OA chondrocytes model was induced via treatment with IL-1β for 24 h. Then the cells were treated with O_3_ and GW9662, the inhibitor of PPARγ. Cell viability was assessed by CCK-8. Further, the cells subjected to Western blot analysis, qRT-PCR and immunofluorescence assay. The numbers of autophagosomes were observed via transmission electron microscopy.

**Results:**

30 μg/ml O_3_ improved the viability of chondrocytes treated with IL-1β. The decreased level of autophagy proteins and the numbers of autophagosomes improved in IL-1β-treated chondrocytes with O_3_ via activating PPARγ/mTOR. In addition, the qRT-PCR results showed that O_3_ decreased the levels of IL-6, TNF-α and MMP-3, MMP-13 in chondrocytes treated with IL-1β.

**Conclusions:**

30 μg/ml O_3_ improved autophagy via activating PPARγ/mTOR signaling and suppressing inflammation in chondrocytes treated with IL-1β.

## Background

With the accelerating aging of the world population, the incidence of degenerative diseases, such as osteoarthritis (OA), is on the rise [1]. OA is the most common joint disorder and one of the most common sources of pain and disability in the elderly. The Global Burden of Disease Study has reported that the global age-standardized point prevalence of OA was 3.75% in 2017, an increase of 9.3% from 1990 [2]. The pathological process of OA includes chronic inflammation and degeneration of cartilage [3]. Researchers have shown that the imbalance in chondrocyte homeostasis is associated with the occurrence and development of OA [4]. Therefore, it is particularly important to keep chondrocytes healthy for preventing the articular cartilage degeneration.

Autophagy is an important protective response of cells exposed to various stresses [5]. It also plays a critical role in articular chondrocytes as the primary mechanism for maintaining normal function and survival. Studies have linked the pathological process of OA with reduced autophagy in chondrocyte [6]. It has been confirmed that improving the level of autophagy can be beneficial in the treatment of OA [7]. In addition, excessive inflammatory cytokines can suppress autophagy activation, causing excessive matrix metalloproteinases (MMPs) expression, leading to cell death [8]. Thus, in the pathological process of OA, inflammatory cytokines also play a vital in cartilage degeneration.

Peroxisome proliferator-activated receptor *γ* (PPAR*γ*) is a nuclear receptor involved in the regulation of many cellular processes [9, 10]. Some studies have indicated that PPARγ activation reduces interleukin (IL)-1-induced expression of inflammatory mediators and MMPs in chondrocytes [11]. PPARγ suppression by diacerein can alleviate oxidative stress and osteoarthritis in mice [12]. PPARγ can also be expressed in different cell types and has been linked to autophagy in other diseases [13, 14]. Vasheghani et al. [15] demonstrated that PPARγ plays a protective role in chondrocytes by regulating the mTOR autophagy signaling pathway. In addition, it was testified that PPARγ is related to autophagy by interacting with the AKT/mTOR pathway. The study showed when AGEs were induced, loss of PPARγ resulted in increased expression of mTOR, PPARγ correlates with phosphorylation of mTOR and PPARγ level was inversely proportion to the phosphorylation in human chondrocytes [16]. Thus, PPARγ may play an important part in the development of OA.

O_3_ was isolated for the first time in 1839 and used as a powerful oxidant in medicine for more than 150 years [17]. In clinical treatment, O_3_ can be applied as a gas mixture of oxygen-ozone (medical- ozone), with different concentration [18–20]. O_3_ can produce free radicals, such as reactive oxygen species (ROS) and lipid hydroperoxides (LOPs) when dissolved in the plasma/serum. The moderate oxidative stress caused by ROS induced an assortment of antioxidant enzymes concentration levels increased, such as superoxide dismutase, glutathione peroxidase, catalase, and NADPH quinone-oxidoreductase [21–23]. Some of these enzymes act as free radical scavengers which were testified to relevant to a wide variety of diseases clinically. It was reported that O_3_ can activate antioxidant protection systems to attenuate chronic constriction injury (CCI)-induced neuropathic pain [24]. In recent decades, O_3_ therapy has been extensively used in clinical practice for treatment of herniated discs, low back pain (LBP) and other chronic pains [25, 26]. The clinical application of O_3_ therapy also produced good clinical therapeutic effects in the treatment of disc herniation and pain [27]. In addition, studies have found that O_3_ at a suitable concentration can improve chondrocyte autophagy in OA by affecting the expression of mammalian target of rapamycin (mTOR) complexes [28]. A meta-analysis showed that repeating the intra-articular injection using an appropriate concentration of 20-30ug/ml and a volume of 15–20 mL of ozone every 7 days for four consecutive weeks is advisable protocol in reducing pain, which produces an improvement of the symptoms at 1, 3 and 6 months after treatment [29]. Ozone therapy to alleviate the symptoms of chronic knee OA has been recognized by an ever-increasing number of physicians. Unfortunately, the evidence to elaborate specific mechanism of O_3_ treatment is not sufficient, especially for OA. There are prejudices among some therapists toward O_3_ treatment. These factors hindered the application of O_3_ therapy in clinically. Thus, the aim of this study was to investigate whether PPAR*γ*/mTOR signaling affected autophagy in chondrocytes treated with IL-1β following O_3_ treatment. The study may reveal the possible underlying mechanisms of O_3_ treatment in OA.

## Materials and methods

### Ethics statement

The rats were provided by the Experimental Animal Center of Shandong University. All rats were killed by cervical dislocation after the experiment. The processes concerning animal use were in compliance with the relevant regulations of the National Institutes of Health and approved by the Animal Care and Use Committee of Shandong Provincial Hospital affiliated with Shandong University. Great efforts were made to minimize the suffering of conscious animals.

### Isolation and culture of rat chondrocytes

Thirty male Wistar rats within three days of birth were used in the present study. The rats were killed for cervical dislocation under general anesthesia and disinfected with povidone iodine and 75% ethanol for 5 min. The cartilage tissue of the epiphyseal of the knee joint was separated by surgical scissors and washed twice with PBS. Then the knee cartilage was cut to about 1 mm^3^. The cartilage was digested with 0.25% trypsin containing ethylenediaminetetraacetic acid (EDTA) for 30 min and 0.2% type II collagenase for overnight at 37 °C. The following day, the digested cells were passed through a 75 μm filter and centrifuged at room temperature at 800 rpm for 4 min. Finally, the chondrocytes were incubated in culture flasks with 10% fetal bovine serum in DMEM/F12 medium containing 10% fetal bovine serum (FBS) added. In this study, chondrocytes from different individual rats were cultured together, it is deficient in cell homogeneity compared with single animal chondrocyte culture, and there may be differences in cell states from different individual animals, which may have some influences on the results but is not decisive.

### Experimental groups and drug administration

IL-1β is a highly potent inducer of cartilage degradation, causing profound loss of proteoglycan from cartilage in vitro and in vivo which was deemed to disrupt the balance between catabolism and anabolism activity [30, 31]. IL-1β-treated chondrocytes or cartilage tissues have been widely adopted as in vitro models to study OA. Then, the chondrocytes were treated with IL-1β (10 ng/mL) (PeproTech) for 24 h in this present study. The incubated cells were randomly divided into the following groups: Control group, IL-1β group, IL-1β + O_3_ group, IL-1β + O_3_ + 3MA group, IL-1β + O_3_ + GW9662 group and IL-1β + GW9662 group. After being treated with IL-1β for 24 h, the cells were exposed to of O_3_ for 30 min at a certain concentration which measured using the O_3_ analyzer purchased from Perma Pure Inc (model MD-050-12-f-4, Perma Pure Inc). Then cells were pretreated with the 3-methyladenine (3MA, 100 nM) and PPARγ inhibitor GW9662 for 12 h prior to O_3_ treatment.

### Cell viability assay

The viability of cells was assessed using cell counting kit-8 assay (CCK-8 kit) (Dojindo Laboratories). Briefly, chondrocytes (6000/well) were seeded in 96-well plates and incubated at 37 °C for 24 h. After being treated with IL-1β for 24 h, the cells were exposed to O_3_ at concentrations of 30, 50 or 70 μg/mL for 30 min. Finally, 10μL of CCK-8 kit solution was added to each well and plates were incubated at 37 °C for 2 h. Absorbance was measured at 450 nm using a microplate reader.

### Western blot analysis

All proteins were extracted using a radio immunoprecipitation assay (RIPA) lysis buffer with protease and phosphatase inhibitors. The protein concentration was measured using a bicinchoninic acid protein assay kit (Beyotime, Shanghai, China). Protein samples were separated using SDS-polyacrylamide gel electrophoresis (SDS-PAGE) and transferred to polyvinylidene difluoride (PVDF) membranes. The membranes were blocked with 5% non-fat milk solution for 1 h at room temperature. The membranes were then probed with anti-PPARγ (sc-7273, 1:200, Santa), anti-mTOR (#2983, 1:1000; Cell Signaling Technology), p-mTOR (#5536, 1:1000; Cell Signaling Technology), anti-ULK1 (#8054, 1:1000; Cell Signaling Technology), anti-LC3II (ab48394, 1:2000, Abcam), anti-P62 (ab56416, 1:2000; Abcam) and anti-β-actin (1:2000; Zhongshan Golden Bridge Biotechnology) overnight at 4 °C. The following day, membranes were washed three times with TBST and incubated with goat anti-rabbit or goat anti-mouse antibody IgG (H + L)-HRP (1:5000; Wuhan Sanying) for 1 h at room temperature. Finally, the membranes were visualized using the enhanced chemiluminescence substrate LumiGLO (Millipore, MA, USA).

### Quantitative real-time PCR (qRT-PCR)

Total RNA was extracted from chondrocytes using Ttizol reagent (Takara, Shiga, Japan). Complementary DNA (cDNA) was amplified using a PrimeScript RT reagent kit (RR047A; Takara). mRNA expression was measured with qRT-PCR using a SYBR® GrenER™ SuperMix (Takara) with the following specific primers for β-actin: forward, 5′-GGGAAATCGTGCGTGAC-3′ and reverse 5′-AGGCTGGAAAAGAGCCT-3′; IL-6: forward, 5′-ATTGTATGAACAGCGATGATGCAC-3′, and reverse 5′-CCAGGTAGAAACGGAACTCCAGA-3′; TNF-α: forward 5′-TTCCAATGGGCTTTCGGAAC-3′ and reverse 5′-AGACATCTTCAGCAGCCTTGTGAG-3′;MMP-13: forward 5′-TGATGATGAAACCTGGACAAGCA-3′ and reverse 5′-GAACGTCATC ATCTGGGAGCA-3′; MMP-3: forward 5′-TGATGGGCCTGGAATGGTC-3′ and reverse 5′-TTCATGAGCAGCAACCAGGAATAG-3′. *β*-actin was used as the internal control, and the level of gene expression was analyzed using the 2^−ΔΔCt^ method.

### Toluidine blue staining

Cells were fixed with 10% neutral buffered formalin at 25 °C for 10 min, washed with distilled water, incubated with 0.05% toluidine blue solution (Sigma, USA) for 30 min at 25 °C, and washed 3 times with distilled water. The stained chondrocytes were observed using the inverted phase contrast microscope.

### Immunofluorescence assay

The cells were fixed with 4% paraformaldehyde for 30 min. Before blocking with goat serum, the cells were permeabilized with 0.3% Triton X-100. The chondrocytes were then incubated with anti-LC3 (ab48394, 1:100, Abcam) and anti-P62 (ab56416, 1:100; Abcam) overnight at 4 °C. The following day, cells were incubated with secondary antibody (1:200; Wuhan Sanying) at room temperature for 1 h. Nuclei were stained with 4′,6-diamidino-2-phenylindole (DAPI) and mounted with anti-fade medium. The stained chondrocytes were observed using immunofluorescence microscopy.

### Transmission electron microscopy (TEM)

The cells were then gathered using a cell scraper and centrifuged to form cell clumps. The cell clumps were fixed with ice-cold 3% glutaraldehyde in 0.1 M cacodylate buffer, post-fixed in osmium tetroxide, embedded in Epon epoxy resin. The samples were then cut into ultrathin sections and further stained using uranyl acetate and lead citrate. Finally, the ultrathin sections were viewed using transmission electron microscopy.

### Statistical analysis

SPSS 22.0 software (IBM Corp. Armonk, NY, USA) was applied for data analysis. Measurement data were described as mean ± standard deviation (SD). The differences among multiple groups were analyzed using one-way analysis of variance (ANOVA), followed by the least significant difference (LSD) test for two-group comparisons among the multiple comparisons. The *P* < 0.05 was considered to indicate a statistically significant difference. All measurements were carried out at least three times.

## Results

### Identification of chondrocytes

**Fig. 1:**
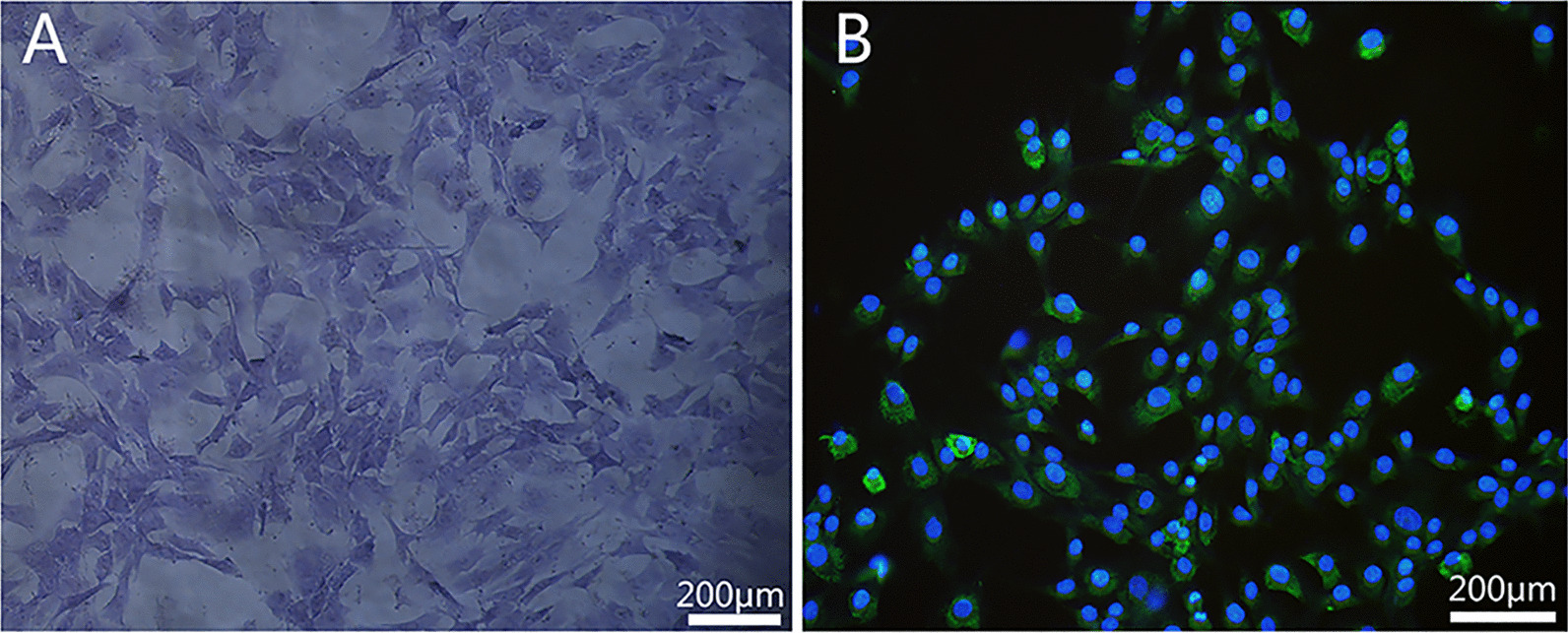
Rat chondrocytes morphological observations. **A** Toluidine blue staining chondrocytes were observed under the inverted phase contrast microscope at magnifications of 20×; **B** More than 95% of the cultured cells showed positive expression of type II collagen under a fluorescence microscope at 20×magnification

Toluidine blue staining result showed the cultured cells are long spindle shaped. The cell nucleus is round and regular, and the cytoplasm is purple-red with a few blue-purple hetero chromatic particles, indicating that the cells were chondrocytes (Fig. 1A). To further identify the isolated cells and determine cell purity, we performed immunofluorescence staining of type II collagen, which is specifically expressed in chondrocytes. The result indicated that more than 95% of the cultured cells were positive for collagen II staining, supporting the above conclusion (Fig. 1B).

### O_3_ (30 μg/ml) improves IL-1β-treated chondrocyte viability

**Fig. 2:**
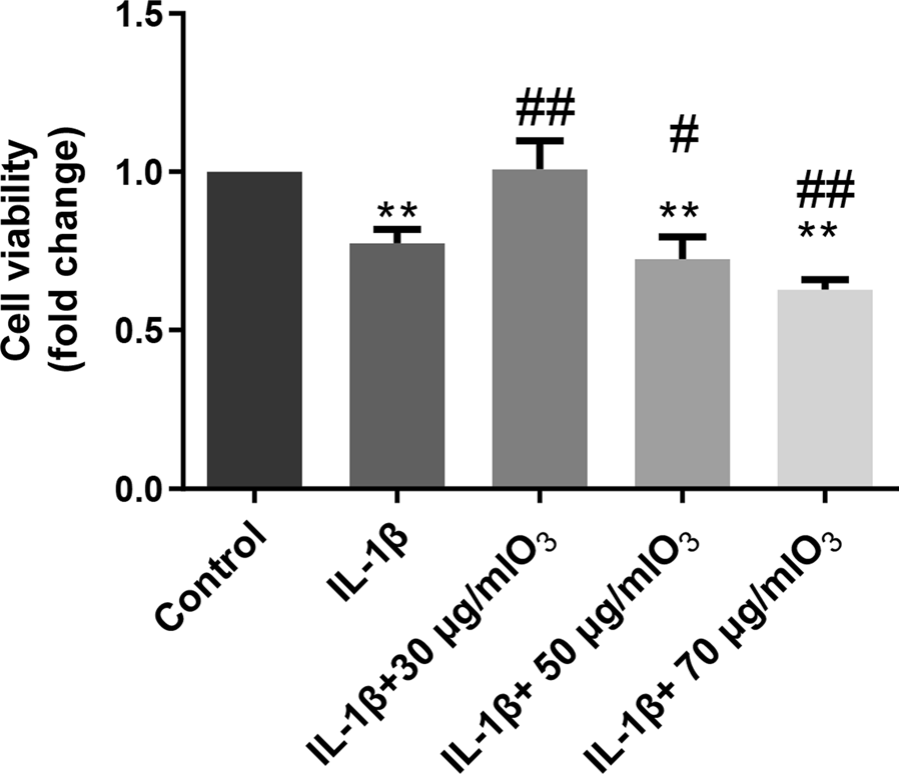
O_3_ treatment improved the cell viability at 30 μg/mL concentration. Chondrocytes were treated with IL-1β (10 ng/mL) for 24 h, then were dealt with different concentration of O_3_ for 30 min.*P < 0.05, **P < 0.01 versus Control group; ^#^*P* < 0.05, ^##^*P* < 0.01 versus IL-1β group

As shown in Fig. 2, 30 μg/ml O_3_ improved cell viability, while 50 and 70 μg/ml O_3_ obviously decreased cell viability relative to treatment with IL-1β alone. Thus, the dose of 30 μg/mL O_3_ was selected for further experiments.

### O_3_ increases the expression of autophagy in chondrocytes treated with IL-1β

**Fig. 3:**
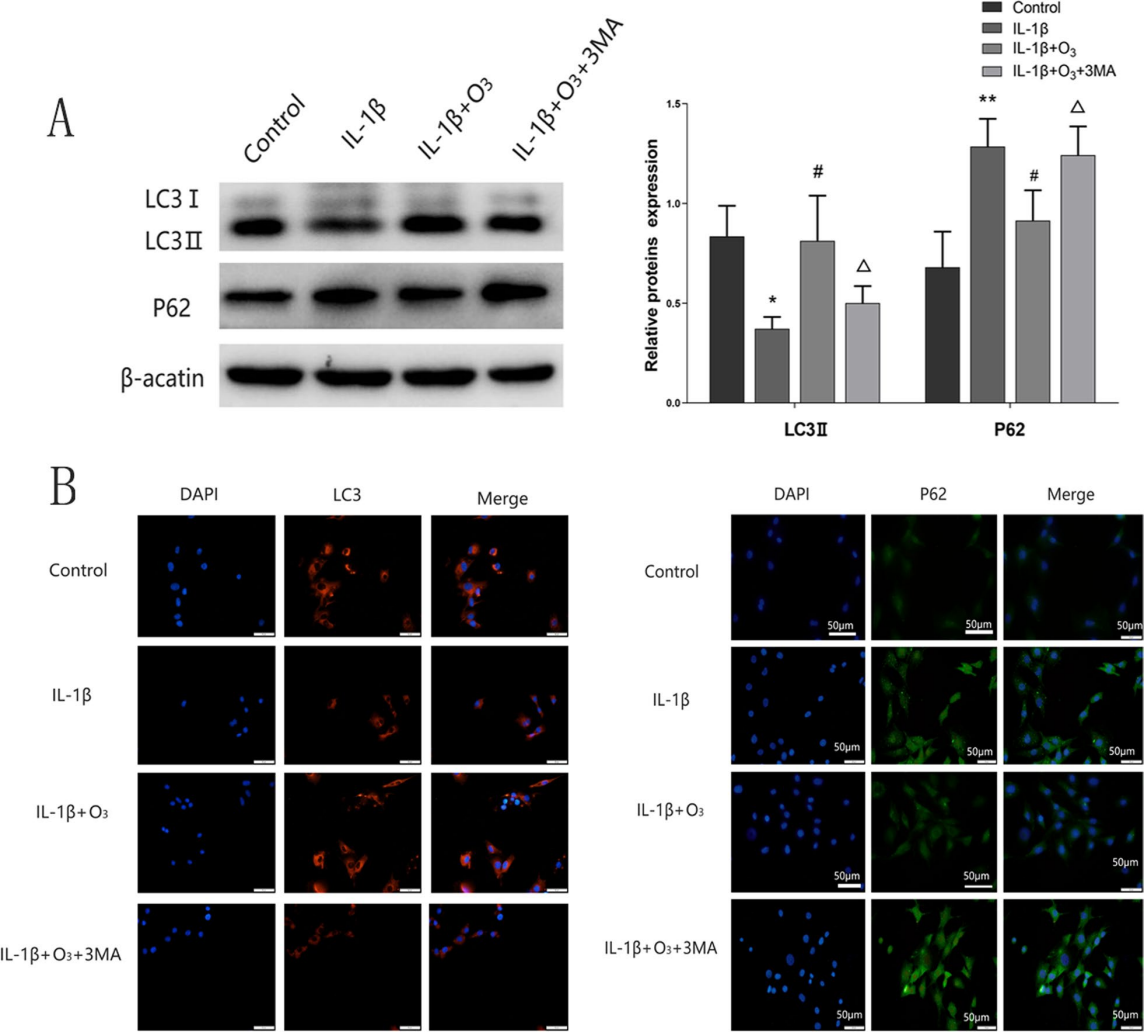
O_3_ increased the level of autophagy of chondrocytes treated with IL-1β. **A** Western blot for the protein expression of LC3 II, P62 and β-actin in each group. **B** The levels of LC3 II and P62 were measured by immunofluorescence at magnifications of 40×. *P < 0.05, **P < 0.01 versus Control group; ^#^*P* < 0.05, ^##^*P* < 0.01 versus IL-1β group; ^Δ^*P* < 0.05, ^ΔΔ^*P* < 0.01 versus IL-1β + O_3_ group

The IL-1β-stimulated chondrocytes were pretreated with the autophagy inhibitor 3MA (100 nM) before treatment with 30 µg/mL ozone. As shown in Fig. 3A, IL-1β administration significantly attenuated the level of LC3 II and increased P62 in chondrocytes. When compared with IL-1β group, the autophagy marker proteins LC3 II obviously elevated and P62 declined in chondrocytes co-treated with IL-1β and O_3_. But O_3_ did not obviously change the low level of autophagy in IL-1β-stimulated chondrocytes pretreated with 3MA (Fig. 3A). As shown in Fig. 3B, the result of immunocytochemical staining shows that compared with the IL-1β group, fluorescence intensities of LC3 enhanced while P62 decreased with O_3_ treatment. While treated with 3MA, accordingly, led to reversed trends.

### O_3_ ameliorates autophagy via PPARγ/mTOR signaling in chondrocytes treated with IL-1β

**Fig. 4:**
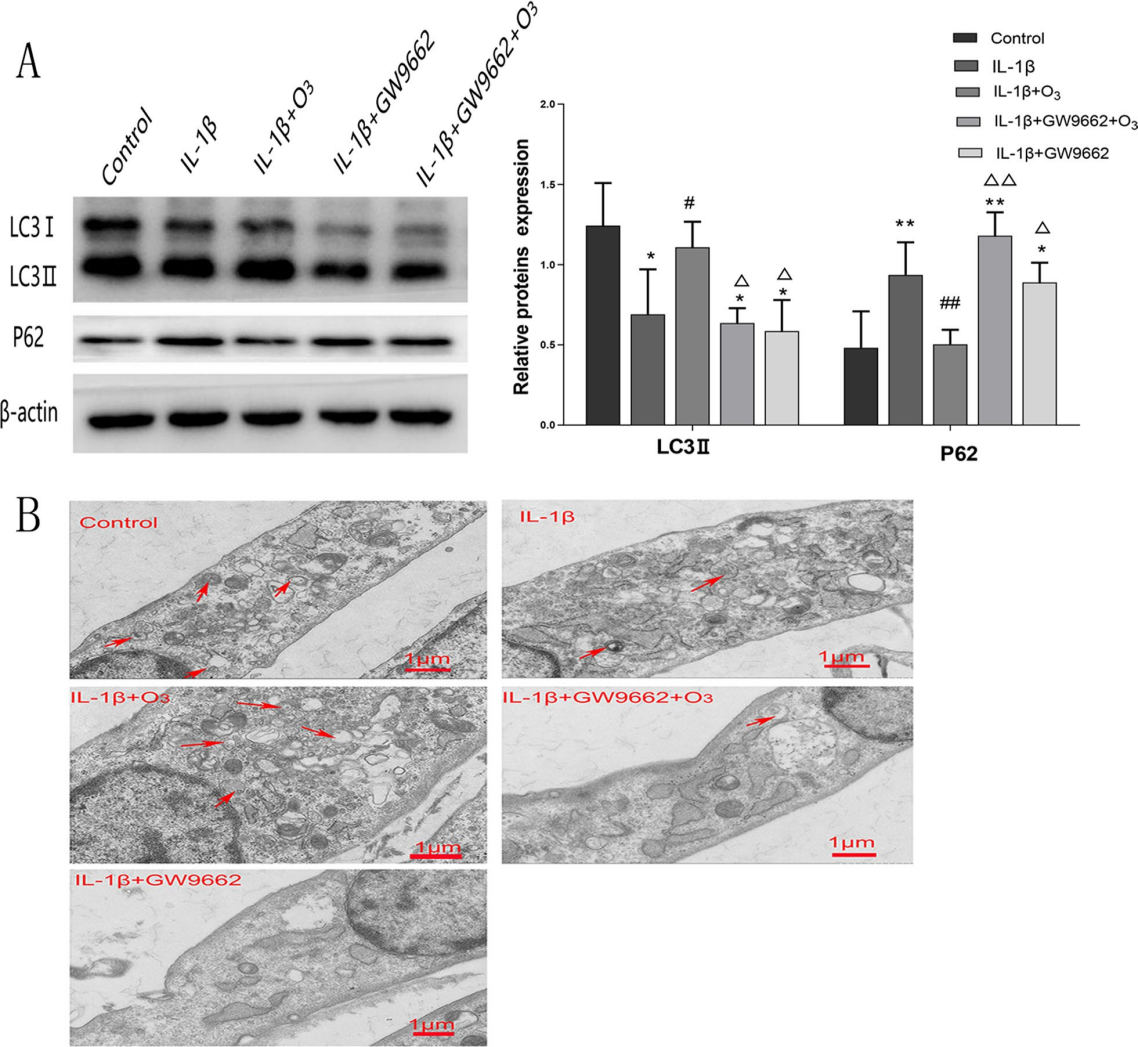
O_3_ increased autophagy of chondrocytes treated with IL-1β but this reversed by GW9662. A. Western blot for the protein expression of LC3 II, P62 and β-actin in each group; B. The numbers of autophagosomes in chondrocytes. The arrows indicate the autophagosomes. *P < 0.05, **P < 0.01 versus Control group; ^#^*P* < 0.05, ^##^*P* < 0.01 versus IL-1β group; ^Δ^*P* < 0.05, ^ΔΔ^*P* < 0.01 versus IL-1β + O_3_ group

The expression of PPARγ has been associated with autophagy. We detected the expression of biomarkers of autophagy in chondrocytes pretreated with GW9662 by Western blotting. The results showed that compared with the IL-1β + O_3_ group, the level of LC3 II decreased and P62 increased in chondrocytes pretreated with GW9662 (Fig. 4A). Besides, TEM analysis revealed that the number of autophagosomes was decreased in chondrocytes treated with IL-1β and improved by O_3_ treatment. In addition, the number of autophagosomes was decreased following treatment with GW9662 (Fig. 4B).

**Fig. 5:**
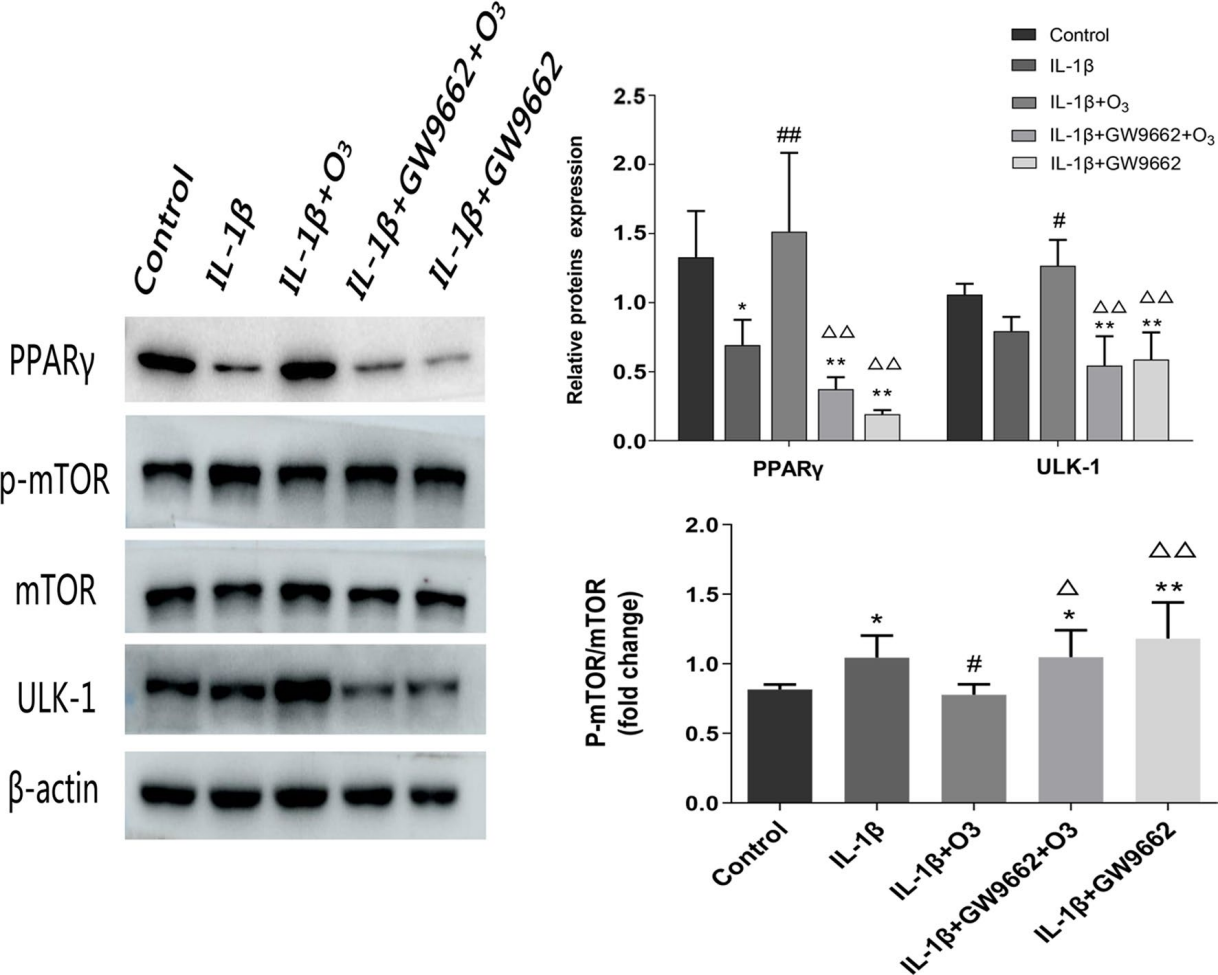
PPARγ/mTOR signaling is involved in O_3_-induced autophagy in chondrocytes treated with IL-1β. Western blot for the protein expression of PPARγ, p-mTOR, mTOR, ULK-1 and β-actin in each group. *P < 0.05, **P < 0.01 versus Control group; ^#^*P* < 0.05, ^##^*P* < 0.01 versus IL-1β group; ^Δ^*P* < 0.05, ^ΔΔ^*P* < 0.01 versus IL-1β + O_3_ group

To further investigate the mechanism of O_3_-induced autophagy in chondrocytes treated with IL-1β, the protein levels of phosphorylated mTOR (p-mTOR), ULK1 were assessed after treated with GW9662. The results of showed that O_3_ decreased p-mTOR and improved the levels of ULK1 in IL-1β + O_3_ group, compared with IL-1β group. In the presence of GW9662, the level of ULK1 was declined significantly and the level of p-mTOR was up-regulated (Fig. 5).

### O_3_ decreases the inflammatory response in chondrocytes treated with IL-1β

**Fig. 6:**
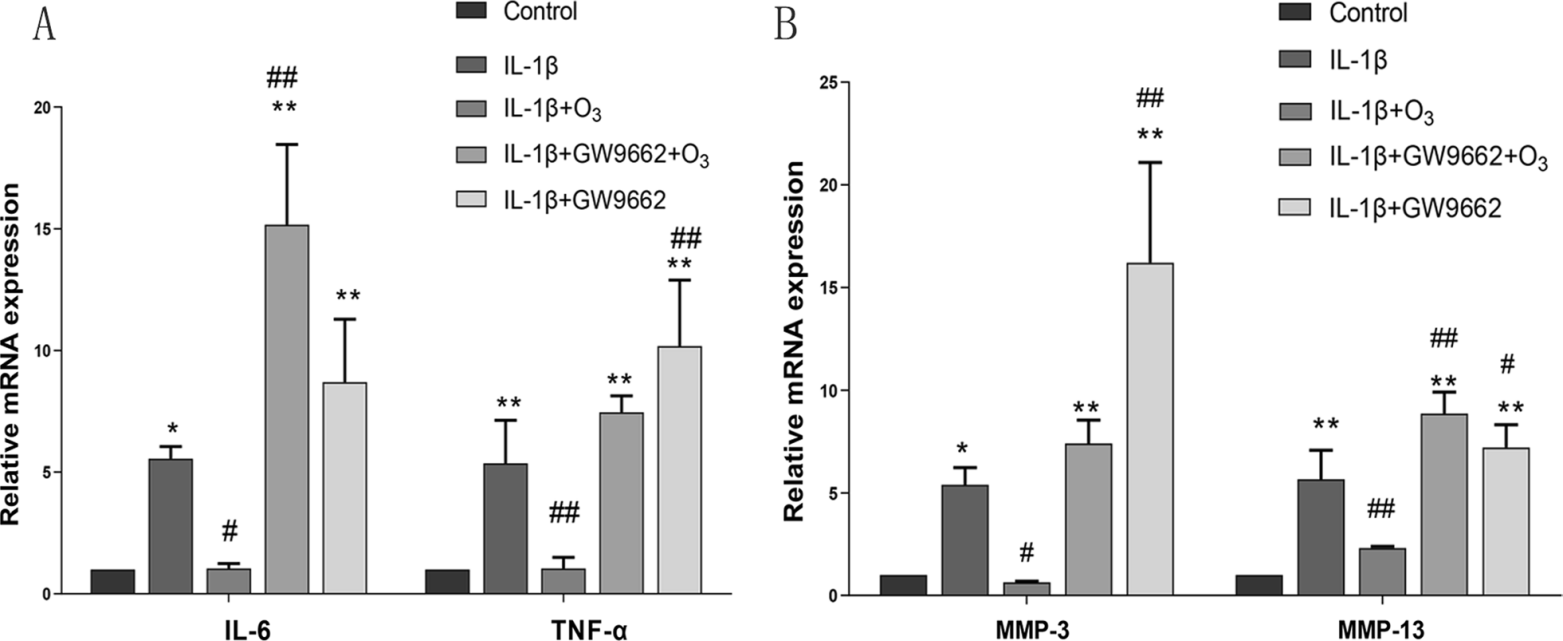
O_3_ decreased the mRNA levels of IL-6, TNF-α and MMP-3, MMP-13 in chondrocytes treated with IL-1*β*. A qRT-PCR for the mRNA expression of IL-6 and TNF-α in each group; B qRT-PCR for the mRNA expression of MMP-3 and MMP-13 in each group. *P < 0.05, **P < 0.01 versus Control group; ^#^*P* < 0.05, ^##^*P* < 0.01 versus IL-1β group; ^Δ^*P* < 0.05, ^ΔΔ^*P* < 0.01vs. IL-1β + O_3_ group

There are various inflammatory cytokines involved in the pathologic process of OA. In this study, the levels mRNA of IL-6, TNF-α, MMP-3, and MMP-13 were measured by qRT-PCR. The results indicated that O_3_ decreased the level mRNA of IL-6, TNF-α, MMP-3 and MMP-13 in IL-1β + O_3_ group, compared with IL-1β group. While treated with GW9662, the level mRNA of IL-6, TNF-α, MMP-3 and MMP-13 was up-regulated significantly (Fig. 6).

## Discussion

OA is a multifactorial disease that is the result of the interaction of mechanical and biological factors [32]. In the progression of OA, inflammatory cytokines such as IL-1β and TNF-α play important roles in promoting the degradation of the cartilage matrix and articular cartilage [32]. IL-1β can induce the expression of many cytokines, changing the expression of various proteases related to the progression of OA [33]. Hence, the chondrocytes treated with IL-1β can be used to simulate OA chondrocytes in vitro studies [34, 35]. Autophagy is a beneficial pathway for sustaining intracellular homeostasis by degrading damaged organelles and long-lived proteins. Previous reports have demonstrated that autophagy may be constitutively activated in normal chondrocytes and defective autophagy in chondrocytes is associated with the degradation of cartilage and the development of OA [36, 37]. Besides, it has been shown that O_3_ can upregulate the reduced autophagy in OA chondrocytes and have beneficial effects in the treatment of OA in clinically [38]. Based on the above, the aim of this study was to investigate the potential molecular biological mechanisms of O_3_ on autophagy in chondrocytes treated with IL-1β.

In this study, we investigated the appropriate concentration of O_3_ for chondrocytes treated with IL-1β using a CCK-8 kit. The results demonstrated that 30 μg/mL O_3_ improved cell viability. Thus, a dose of 30 μg/mL O_3_ was chosen to the treatment of chondrocytes treated with IL-1β. In the present study, the results indicated that IL-1β suppressed the level of autophagy which was reflected by reduced LC3II and increased P62 in chondrocytes. LC3 conversion (LC3-I to LC3-II) reflects the progression of autophagy, and detecting LC3 by immunoblot analysis is often used to monitor autophagic activity [39]. The p62 protein, also called sequestosome 1 (SQSTM1), binds directly to LC3 via a short LC3 interaction region, which degraded by autophagy and served as a marker to study autophagic flu [40]. After treatment with O_3_ (30 μg/mL), the levels of autophagy were enhanced in IL-1β-treated chondrocytes. But the ozone-induced autophagy was inhibited by 3MA, which is commonly used as autophagy inhibitor, selectively inhibiting class III PI3K to block autophagic activation. These findings indicate that O_3_ regulates autophagy in IL-1β-treated chondrocytes.

Previous studies have shown that the activation of PPARγ has beneficial effects on the level of autophagy in type 2 diabetic mice. In addition, deletion of the PPARγ gene can lead to cartilage-specific destruction leading to spontaneous OA [15].To further explore the role of PPARγ in O_3_-induced autophagy, chondrocytes were treated with the PPARγ inhibitor GW9662 before O_3_ treatment. The potent, irreversible and selective PPARγ antagonist GW9662 was proved to prevent the activation of PPARγ and inhibit growth of human mammary tumor cell lines [41]. In this study, we found that the level of PPARγ is sharply reduced following treatment with GW9662. Besides, the levels of LC3II decreased while the levels of P62 increased following treatment with GW9662. The O_3_-induced autophagy was reversed by GW9662. Considering the negative effect of GW9662 on autophagy, it is logical that PPARγ plays a key role in O_3_-induced autophagy. Furthermore, the results of TEM on autophagosomes also elucidate this issue well.

As PPARγ could induce aberrant mTOR signaling in chondrocytes, we suppose that in O_3_-treated chondrocytes, PPARγ acts via the mTOR/autophagy signaling pathway. mTOR is the target molecule of rapamycin, which is the point of attachment between autophagy and the upstream signaling pathway. mTOR is usually considered a suppressor of autophagy and is involved in the inhibition of autophagy in chondrocytes, causing cartilage damage in OA [42]. Moreover, our data demonstrate that GW9662 reversed the O_3_-mediated suppression of the phosphorylation of mTOR. ULK1, a homolog of yeast ATG1, is another key protein regulating autophagy. A previous study reported that ULK1 expression is negatively regulated by mTOR or activated directly by AMPK [43]. It was identified in HEK 293 cells where down-regulation of ULK1 was sufficient to inhibit autophagy [44]. Our data shows that ULK1 levels significantly decreased following an increase in p-mTOR in chondrocytes treated with GW9662. Therefore, the PPARγ/mTOR pathway is activated in the chondrocytes in this study. But the mechanism of ozone action has not been clarified yet, and the study has shown that inhibiting the vicious cycle of TNFα-syndecan 4-TNFα may also play important roles in chondrocyte autophagy [45]. Therefore, ozone could induce autophagy in part by activating PPARγ/mTOR signaling.

Since inflammation plays an important role in the development and progression of OA, the development of therapies focusing on inhibiting inflammation is increasing. According to a study of inflammatory cytokines, the result indicated that some cytokines, such as TNF-α and IL-6, accelerated the progression of OA by promoting the catabolism of cartilage [46]. Certain studies have demonstrated that IL-1β could suppress the autophagy pathway in chondrocytes [47]. Importantly, activation of PPARγ has been shown to have beneficial effects in alleviating inflammation in human OA chondrocytes [48]. In the present study, we found that O_3_ inhibits the mRNA levels of the TNF-α and IL-6 in chondrocytes of treated with IL-1β. However, this result was reversed by the suppression of PPARγ. In addition, a number of studies have indicated that activation of PPARγ can inhibit NF-κB activation and decrease the levels of inflammatory cytokines [49].The activation of autophagy can be suppressed by excessive release of inflammatory cytokines [50]. Considering that O_3_ blocked IL-1β-induced autophagy inhibition in chondrocytes, the result is likely due to its anti-inflammatory effect on chondrocytes. Moreover, study has shown that IL-1β can induce the expression of MMPs, in turn inducing cartilage matrix degradation [51]. The result of qRT-PCR results revealed that the mRNA levels of MMP-13 and MMP-3 were decreased with O_3_ treatment. These data suggest that inhibition of inflammatory cytokines through activation of PPARγ has beneficial effects on IL-1β-treated chondrocytes. Our findings suggest O_3_ seems to be able to attenuated the IL-1β-induced production TNF-α, IL-6, and MMPs. Taken together, O_3_ PPARγ activation plays an important role in the protective effect of O_3_ in chondrocytes treated with IL-1β.

There were some limitations to our study. We did not test whether administering PPARγ agonist treatment had the protective effects in chondrocytes treated with IL-1β. In this study, we observed the PPARγ pathways is involved in the protective effects of osteoarthritis by reducing inflammatory reaction. However, the specific effects of these pathways in osteoarthritis are worth pursuing. In addition, the specific effects of these pathways in vivo remain to be investigated.

## Conclusions

In this study, we revealed that O_3_ can induce autophagy by activating the PPAR*γ*/mTOR signaling pathway and inhibit the inflammatory cytokines via the activation of PPAR*γ* in chondrocytes treated with IL-1β.

## Data Availability

All the data generated or analyzed during this study are included in this published article.

## Abbreviations

OA: Osteoarthritis
MMPs: Matrix metalloproteinases
PPARγ: Peroxisome proliferator-activated receptor *γ*
O_3_: Ozone
ROS: Reactive oxygen species
LOPs: Lipid hydroperoxides
mTOR: Mammalian target of rapamycin
FBS: Fetal bovine serum
qRT-PCR: Quantitative real-time PCR
TEM: Transmission electron microscopy
ANOVA: One-way analysis of variance
